# Measuring and Monitoring Progress Toward Health Equity: Local Challenges for Public Health

**DOI:** 10.5888/pcd11.130440

**Published:** 2014-09-18

**Authors:** Snehal N. Shah, Elizabeth T. Russo, Tara R. Earl, Tony Kuo

**Affiliations:** Author Affiliations: Elizabeth T. Russo, Boston Public Health Commission, Boston, Massachusetts; Tara R. Earl, ICF International, Inc, Atlanta, Georgia; Tony Kuo, Los Angeles County Department of Public Health and UCLA David Geffen School of Medicine, Los Angeles, California. Dr Shah is also affiliated with Boston University School of Medicine, Boston, Massachusetts.

## Abstract

To address health disparities, local health departments need high-resolution data on subpopulations and geographic regions, but the quality and availability of these data are often suboptimal. The Boston Public Health Commission and the Los Angeles County Department of Public Health faced challenges in acquiring and using community-level data essential for the design and implementation of programs that can improve the health of those who have social or economic disadvantages. To overcome these challenges, both agencies used practical and innovative strategies for data management and analysis, including augmentation of existing population surveys, the use of combined data sets, and the generation of small-area estimates. These and other strategies show how community-level health data can be analyzed, expanded, and integrated into existing public health surveillance and program infrastructure to inform jurisdictional planning and tailoring of interventions aimed at achieving optimal health for all members of a community.

## Introduction

Public health surveillance data often lack the geographic resolution to sufficiently describe or make statistical inferences about certain subpopulations and geographic areas. Because of this limitation, local health agencies must rely on data for populations that differ from their constituent communities (1–4). As a result, local agencies have limited capacity to detect and monitor the health of subpopulations that are more affected by chronic disease than the general population (3–6). Health equity, or optimal health for all, and the reduction of health disparities are achievable if local health data are available to offer finer resolution for strategy planning (7).

The Institute of Medicine recommends investing resources in disease surveillance systems that provide granular data to inform efforts designed to reduce health disparities among subpopulations (8). Similarly, the 2010 Patient Protection and Affordable Care Act (ACA) mandates federally conducted public health programs, activities, and surveys to collect and report data on race, ethnicity, and primary language at the “smallest geographic level” possible (9). Although the need for granular data is widely recognized, local health departments continue to face barriers to collecting or compiling such data (3–6). Without further investment in strategies to overcome these barriers, local health departments are constrained in their ability to plan and allocate the appropriate resources to address community needs. In this article, we highlight examples of practical, applicable strategies undertaken by 2 local health departments to acquire and compile granular data needed for local public health planning and program development.

## Challenges and Strategies

Boston, Massachusetts, and Los Angeles County, California, 2 jurisdictions with diverse populations (Table 1), have prioritized health equity as an achievable public health goal. Racial/ethnic minority populations in Boston and Los Angeles County, like racial/ethnic minority populations in other urban settings in the United States, have high rates of chronic diseases compared with whites (Table 2). Both the Boston Public Health Commission (BPHC) and the Los Angeles County Department of Public Health (LACDPH) received federal and state funding to reduce health disparities among populations at high risk for poor health outcomes (eg, racial/ethnic minority groups, the elderly, the homeless). To find data-driven solutions, each agency found ways to overcome challenges to acquiring and compiling subpopulation data required for high-quality surveillance, programming, and program evaluation. We identified 4 key challenges and several strategies for each (Table 3).

**Table 1:** Population Characteristics in Boston and Los Angeles County, 2010^a^^,^ b

Characteristic	Boston, n (%)	Los Angeles County, n (%)
**Total population**	617,594	9,818,605
**Age, y**		
≤19	135,592 (22.0)	2,711,958 (27.7)
20–64	419,765 (67.9)	5,288,160 (61.5)
≥65	62,237 (10.1)	1,065,699 (10.9)
**Sex**		
Female	321,643 (52.1)	4,978,951 (50.7)
**Race/ethnicity**		
Black, non-Hispanic/non-Latino	138,073 (22.4)	815,086 (8.3)
Hispanic/Latino	107,917 (17.5)	4,687,889 (47.7)
White, non-Hispanic/non-Latino	290,312 (47.0)	2,728,321 (27.8)
Asian/Pacific Islander	55,028 (8.9)	1,348,135 (13.7)
Other	26,264 (4.3)	239,174 (2.5)
**Education (for population aged ≥25 y)**		
High school/equivalent or less	150,592 (38.0)	2,828,114 (44.7)
Some college or college degree	245,863 (62.0)	3,490,191 (55.3)
**Median household income, 2011 inflation-adjusted $**	51,739	56,266

a All values are numbers (percentages) unless otherwise indicated.

b Source: US Census (10,11).

**Table 2:** Life Expectancy and Prevalence of Adult Obesity, Hypertension, Diabetes, and Smoking, by Race/Ethnicity in Boston and Los Angeles County^a^

Characteristic	Life Expectancy, y	Obesity, % (95% CI)	Hypertension, % (95% CI)	Diabetes, % (95% CI)	Smoking, % (95% CI)
**Boston**					
Asian	88.8	9.4 (1.0–17.8)	17.9 (7.6–28.2)	—b	4.7 (0.2–9.3)
Black, non-Hispanic/non-Latino	76.8	33.0 (28.0–38.0)	31.5 (27.3–35.6)	9.3 (7.3–11.3)	14.3 (10.9–17.7)
Hispanic/Latino	88.4	24.8 (19.1–30.5)	20.7 (16.1–25.4)	7.4 (5.0–9.8)	14.5 (9.8–19.2)
White, non-Hispanic/non-Latino	78.9	15.7 (13.7–17.8)	21.7 (19.7–23.7)	4.9 (4.0–5.8)	17.6 (15.3–19.9)
**Los Angeles County**					
Asian	84.4	8.9 (6.3–11.5)	25.0 (21.3–28.8)	9.3 (7.0–11.6)	9.2 (6.5–11.9)
Black, non-Hispanic/non-Latino	73.8	31.0 (26.8–35.2)	39.2 (34.8–43.7)	12.6 (9.9–15.4)	17.2 (13.6–20.7)
Hispanic/Latino	81.6	31.6 (29.3–34.0)	18.0 (16.2–19.7)	9.5 (8.2–10.8)	11.9 (10.2–13.7)
White, non-Hispanic/non-Latino	79.3	18.0 (16.2–19.8)	27.4 (25.5–29.2)	8.5 (7.3–9.8)	15.2 (13.4–17.1)

Abbreviation: CI, confidence interval.

a Data on life expectancy for Boston are for combined years 2006–2010: Boston Public Health Commission (12). Data on life expectancy in Los Angeles are for 2007 only: Los Angeles Department of Public Health (13). Data on prevalence of obesity, hypertension, diabetes, and smoking in Boston are for 2010 only: Boston BRFSS (14). Data on prevalence of obesity, hypertension, diabetes, and smoking in Los Angeles are for 2010–2011: Los Angeles County Department of Public Health (15).

b Insufficient sample size.

**Table 3:** Challenges to Integrating a Health-Equity^a^ Perspective in Local Disease Surveillance and Health Assessment: Corresponding Strategies for Strengthening Data Collection and Data Analysis

Data-Related Challenges	Strategies for Addressing Challenges
**Challenge no. 1: Variables that identify differences in population health are often not collected.** Existing national surveillance and data collection systems often contain a finite set of sociodemographic and other variables (eg, race/ethnicity, educational attainment, income). Data on several variables relevant to health equity issues (housing type, employment type, length of residence in United States) are typically not collected or adequately captured by these systems.	Through leveraging of partnerships, shared resources, and extramural funding, add variables of interest to existing systems of data collection.
**Challenge no. 2: Small sample size and sparse population data limit analysis.** Small sample sizes for key subpopulations often limit analysis of surveillance and health assessment data to address health equity issues.	Increase power for these analyses by using such methods as augmenting data collection via oversampling of target groups, combining data sets across multiple years, or conducting small-area estimate analysis to model rates by city or community.
**Challenge no. 3: Inconsistent variable types and variable-response categories.** There is a lack of standardization in data collection (eg, type of questions asked, type of data collected) for key variables and variable-response categories often used to measure disparities.	Integrate standard data collection question modules and/or procedures in existing surveillance and data collection systems and in public health programs by enacting regulatory mandates via state or local governing entities or through addition of requirements in contracts to program vendors or clinics.
**Challenge no. 4: Data needed for certain program planning or evaluation do not exist.** Data for certain program planning and evaluation do not exist and must be collected.	In selected cases, conduct primary data collection on target groups and/or collect primary data for interventions or programs of interest.

a The Centers for Disease Control and Prevention provides the following definition for health equity: “all individuals have the opportunity to attain their full health potential, and no one is disadvantaged from achieving this potential because of their social position or other socially determined circumstance” (16).

### Challenge no. 1: Variables that identify differences in population health are often not collected

State and national systems for surveillance and data collection, such as the Behavioral Risk Factor Surveillance System (BRFSS) and state vital record systems, contain a set of variables on race, Hispanic/Latino ethnicity, income, educational attainment, and other sociodemographic characteristics; these variables vary by survey or data system. Data on additional sociodemographic variables such as ethnicity, housing type (subsidized vs market-rate), type of employment, and length of residence in the United States can strengthen the capacity of these surveillance systems to identify differences in the health of at-risk populations but are often not collected. The absence of these data can result in an incomplete understanding of how health outcomes are associated with local sociodemographic and cultural characteristics.

#### Strategies for adding variables to existing data collection systems

Every 2 years, BPHC conducts a random-digit–dialed, citywide community health surveillance survey of 2,500 to 3,000 Boston residents — the Boston Behavioral Risk Factor Surveillance Survey (Boston BRFSS) (14,17). In 2001, BPHC added a question to assess whether a respondent lived in public housing, subsidized housing, or neither. Adding this question allowed BPHC to monitor the health of vulnerable public housing residents. Residents of public housing in Boston have a significantly higher prevalence of asthma than other city residents have (18). To address this and other health disparities, BPHC, the Boston Housing Authority, and other partners developed interventions to reduce exposure to pests, pesticides, and secondhand smoke among public housing residents. These interventions, and others, contributed to a decline in self-reported asthma symptoms among public housing residents (S.N.S, unpublished data, 2013).

A similar strategy was used by LACDPH to better characterize caregivers in Los Angeles County. In 2007, LACDPH expanded its random-digit–dialed, countywide Los Angeles County Health Survey (LACHS) of 8,000 households to include a series of questions on caregiving for the elderly and disabled (15,19,20). Data on the prevalence and costs of caregiving helped local agencies apply for grants and prioritize resources for their aging populations.

Even with limited funding, many states and local jurisdictions have the capacity to add additional variables to existing population surveys like the BRFSS.

### Challenge no. 2: Small sample size and sparse population data limit analysis

Population surveys may yield small sample sizes that reduce the reliability of survey estimates and limit the ability of local health departments to detect differences in health outcomes by race, ethnicity, and/or other sociodemographic characteristics (5). In jurisdictions like Los Angeles County, which has 88 cities and one large unincorporated area, county-level data on disease prevalence are often insufficient to inform city or community policies or decisions about resource allocations. In this situation, stratified analysis of such health indicators as obesity rates by city or community is required.

#### Strategies to address sample-size limitations

**Augment sample size.** In 2009, BPHC was awarded grants from the Centers for Disease Control and Prevention (CDC) that included funding for administration of the BRFSS in 2010 and 2012 to a grant-specific sample of the Boston population (different from the sample of the Boston BRFSS described previously) consisting of 1,500 city residents (21). Leveraging this opportunity, BPHC used grant funds, which could have been used to support program activities, to expand the sample size to 2,000, a number that, based on a priori power analysis, would allow for more stable point estimates of neighborhood variables important for evaluating the impact of local interventions.

**Combine data years.** The number of completed Boston BRFSS surveys (2,500–3,000) is sufficient for monitoring general population health trends in Boston but lacks power to conduct subgroup analyses in smaller neighborhoods. To mitigate this shortcoming, BPHC generates neighborhood and subgroup estimates by combining survey data from several years. Combining data across years requires consistent survey methodology, survey instruments, and weighting procedures across time.

**Generate small-area estimates.** With multiple municipalities in Los Angeles County, program planning and resource-allocation decisions often require granular, geographically subdivided health data that are not readily available through existing sources. The LACHS provides relatively precise point estimates for many health indicators in larger regions but lacks the sample size and data for producing similarly precise estimates for cities or council districts. Recently, LACDPH applied small-area estimation to generate stratified estimates of obesity and smoking prevalence by city and city council district using data from the LACHS, the US Census, and the LA County Population Estimates and Projection System. Small-area estimation applies statistical procedures to generate a series of dependent variable projections based on regression modeling; the method uses multiple sources of information that provide data not available from a single source (22,23). Small-area estimation has several limitations: 1) the estimates are only as good as the model assumptions and the quality of data used; 2) in many instances, no amount of supplemental information can compensate for extremely small sample sizes; and 3) diagnostics for checking nonlinear models are generally not well developed for this method (23).

### Challenge no. 3: Inconsistent variable types and variable–response categories

Variables and variable–response categories for documenting race and ethnicity, frequently used to measure racial and ethnic health disparities, are often not standardized among institutions and levels of government. Although the federal Office of Management and Budget provides standards for reporting broad categories of race and Hispanic/Latino ethnicity, variables and response categories used to collect these data vary, and institutions may not use updated standards (24–26). Adhering to standardized variable definitions, response categories, and formats to collect sociodemographic data increases the value of the data by making them more relevant, comparable, and useful in multiple settings. Standard variables and response categories may evolve by necessity, but their evolution makes comparisons over time more difficult.

#### Strategies to standardize data collection

**Enact regulatory mandate.** In 2006, Boston’s Board of Health passed a data collection regulation that requires all acute-care hospitals and community health centers in Boston to collect data on race, ethnicity, preferred language, and educational attainment in a standardized format from patient self-report for all patient encounters. The regulation also requires that sociodemographic data for every inpatient, outpatient, and emergency department patient encounter be shared with BPHC. That same year, the Massachusetts Department of Public Health (MADPH) passed a similar statewide regulation. BPHC and MADPH aligned their standards so that institutions in Boston could collect data in a single format to satisfy both local and statewide regulations. Although the new regulations required high levels of technical assistance, training, and coordination among agencies, the newly consistent data will help agencies to understand where racial and ethnic subpopulations in Boston seek medical care.

**Embed requirements in contracts.** In Los Angeles County, data collection is often standardized through the subcontracting process. Wherever feasible, LACDPH embeds standardized data collection requirements in its subcontracts with funded partners. To gauge and identify program gaps in clinical preventive services in the ambulatory care setting, LACDPH includes requirements in subcontracts with clinical organizations, calling for participating clinic networks to collect and provide certain data on patient demographics and clinical indicators.

### Challenge no. 4: Data needed for certain program planning or evaluation do not exist

Using existing data and the aforementioned strategies may still be insufficient for local decision making. Existing data sources may focus on populations that are not relevant to program objectives or have data collection time points that do not align with program timetables. New community data are often required to better tailor interventions to address health disparities in target populations.

Although primary data collection at the population and program levels can be resource-intensive, it can offer high geographic resolution and subpopulation-specific information. Generally, primary data collection requires technical expertise for the design, implementation, and analysis of data sets as well as a sizable financial investment (27). Because most state and local health departments lack continuous capacity to collect primary data, these efforts should be targeted and tailored to achievable program goals.

#### Strategies for collecting new data for program planning and evaluation

**Collect primary health assessment data.** To address health disparities in regions of Los Angeles County that have high rates of poverty and obesity, LACDPH conducted 2 health and nutrition examination surveys (LA HANES) during 2010–2012. These surveys recruited low-income adults who relied on multipurpose public health centers for preventive services, health education, and community programming. The first survey (n ~ 720; response rate, 74%) was conducted during the first 15 months of a regional, 2-year, CDC-funded obesity prevention initiative. The second survey (n ~ 1,500; response rate, 69%) was completed approximately 12 months later. The surveys collected data on self-reported sociodemographic characteristics, self-reported health behaviors, and measured height and weight, waist circumference, blood pressure, and urinary cotinine (28,29).

Preliminary data from LA HANES spurred several policy and program planning discussions, including how LACDPH might prepare for changes in the delivery of public health services as a result of ACA. That nearly half of all black and Hispanic/Latino residents had a blood pressure measurement in the prehypertensive or hypertensive range underscored the need for more tailored approaches to detect and treat this risk factor for cardiovascular disease in these subpopulations (29).

**Collect primary program data.** In 2011, BPHC cosponsored the launch of a new bicycle-share program in Boston with an aim to include residents from low-income neighborhoods as well as the city’s center, which is generally composed of high-income neighborhoods. BPHC leveraged its support to have 4 of the 61 initial stations strategically placed in low-income neighborhoods, and it distributed subsidized program memberships to hundreds of eligible low-income residents.

Registration for program membership was available online through the bicycle-share vendor website or via telephone if Internet was not accessible. To understand the characteristics of program members, BPHC asked the vendor to include a question about race and ethnicity on the online registration page. Although adding this one question necessitated reprogramming on the part of the vendor, the data collected by making this one addition documented the volume of subsidized memberships and demonstrated the increased program reach to black and Hispanic/Latino populations. Among program members who disclosed their racial and ethnic identity, the percentage that self-reported as black or Hispanic/Latino was nearly threefold higher among subsidized members than among nonsubsidized members.

## Considerations

Differential quality and availability of subpopulation data can reduce the ability of local health departments to address health disparities. Because health and sociocultural experiences vary across race, ethnicity, and geographic area, local public health systems require more granular data to understand and reduce disparities to achieve meaningful changes in their communities (5,6,30). Albeit resource- or time-intensive, these strategies have been used by local agencies to optimize pre-existing data sources and guide primary data collection. However, other strategies may still be needed to address the various domains of health disparities. Federal funding, for instance, can require that a fixed percentage of funds be allocated to community surveillance and program evaluation. Complementary grant mechanisms, which support technical assistance and mentoring, can help expand the use of sophisticated analytic methods to examine local health equity issues.

Nonfederal data sources, such as hospital-discharge administrative data, may also be useful for answering health equity questions (26). For example, adding sociodemographic variables to existing standardized systems of clinical data collection can contribute to a better understanding of how social determinants of health affect national, state, and local hospitalization rates. Hospitals may use existing clinical data for their ACA-mandated assessments of community health care needs, but many may choose to partner with local health departments to initiate primary data collection in the community (9).

Although many of the strategies described here have limitations (eg, implementation costs, staffing burden, varying availability of expertise by community), collectively they strengthen local data systems for supporting and achieving optimal health for all. The experiences of Boston and Los Angeles County illustrate how local health departments can strengthen disease surveillance and community-level data collection to build capacity and implement replicable strategies for addressing social and economic disadvantages associated with poor health.
